# Evaluation of Un-Medicated, Self-Paced Alcohol Withdrawal

**DOI:** 10.1371/journal.pone.0022994

**Published:** 2011-07-28

**Authors:** Martin Craig, Antonio Pennacchia, Neil R. Wright, Henry W. Chase, Lee Hogarth

**Affiliations:** 1 Nottingham Substance Misuse Service, Nottingham, United Kingdom; 2 Mental Health, Learning Disabilities and Marginalised Groups, National Health Service (NHS) Nottingham City, Nottingham, United Kingdom; 3 Department of Psychiatry, Western Psychiatric Institute and Clinic, University of Pittsburgh School of Medicine, Pittsburgh, Pennsylvania, United States of America; 4 School of Psychology, University of Nottingham, Nottingham, United Kingdom; University of Minnesota, United States of America

## Abstract

It is currently unclear how effective un-medicated, self-paced alcohol withdrawal is in reducing alcohol consumption in alcohol dependent clients. To address this question, the current study examined the reduction in alcohol consumption, assessed by breath alcohol and drink diary self-report, of 405 alcohol-dependent clients over a 10-day, un-medicated, self-paced alcohol reduction program that included group discussion of strategies for titrating between withdrawal and intoxication. It was found that attendance at treatment sessions was associated with a reduction in alcohol consumption, reflected in both breath alcohol and diary measures, and these two measures were significantly correlated. Overall, 35% of clients achieved a zero breath alcohol reading by their final session, although this percentage increased to 56% of clients who attended all 10 sessions. Withdrawal seizures occurred in only 0.5% of clients despite 17.2% having a history of seizures in other settings. It is concluded that the alcohol reduction protocol outlined here provides an effective and safe method for reducing alcohol consumption in severely alcohol dependent clients, and that methods for augmenting attendance, such as contingency management, should enhance the effectiveness of this treatment.

## Introduction

Many therapeutic approaches to treating alcohol dependence require that client terminate drinking prior to entry. This requirement is often stipulated to facilitate the management of clients, to avoid adverse interactions with pharmacotherapy (for example, benzodiazepines) and to improve clients' engagement with the therapeutic intervention. However, it is often the case that severely alcohol dependent clients find this requirement difficult to comply with, resulting in such clients being assigned into acute medicated detoxification prior to further treatment. However, growing evidence showing that sudden alcohol withdrawal produces lasting neurocognitive dysfunction [1] has highlighted the need for a gradual, self-paced alcohol reduction strategies to prepare clients for treatment entry, whilst negating the damaging consequences of sudden alcohol withdrawal.

There have been a number of documented examples of gradual alcohol reduction with in-patients [2], [3] and out-patients [4], [5]. These studies have reported successful reductions in drinking, although the sample sizes of these studies have tended to be small, and/or the reduction of alcohol consumption across the treatment period has not been recorded systematically. The purpose of the current study was to report data on effectiveness of a 10-day, self-paced, alcohol withdrawal protocol to provide an empirical basis from which to evaluate this method for reducing alcohol consumption.

## Materials and Methods

### Ethics statement

All participants provided informed written consent to participate, and the methods were approved by the Nottinghamshire NHS ethics committee and the University of Nottingham School of Psychology ethics committee, and conformed to the principles expressed in the Declaration of Helsinki.

### Procedure

At Oxford Corner, a facility of the Nottingham Substance Misuse Service, the Gradual Alcohol Reduction Group (GARG) has been running since July 2003 to assist alcohol dependent individuals to self-wean from alcohol prior to more protracted/intensive treatment. Breath alcohol concentrations (BrACs) were recorded between July 2003 and December 2008 from 405 clients during their participation in ten-daily group sessions.

### Participants

Clients were selected for the GARG program on the grounds that their alcohol consumption was too high for sober entry into the therapeutic day hospital programme, or if they reported withdrawal symptoms or early drinking to relieve withdrawal. Specifically, clients who breathalysed high during initial clinical screening but appeared sober were considered appropriate for entry. No upper or lower limit on the amount drunk was set for inclusion, although clients who were unmanageably inebriated or clients who returned a positive BrAC merely because they had drunk shortly before their appointment were reassessed for their suitability at a later point. Clients who drank at the lower range were not included if they were deemed suitable for inclusion in other treatment services, and this was based on clinical judgement rather than a priori criteria. A history of alcohol withdrawal seizures was not an exclusion criterion, nor was existing liver disease. Clients with liver disease were limited, on a case-by-case basis with close monitoring, as to how long they were allowed to participate. Clients with co-existing mental health problems were not excluded but their mental health was monitored and assessed as necessary. Concurrent treatment for drug misuse was not an exclusion criterion. Thus, the study accepted clients on a case by case assessment, with a view to accepting clients that would have difficulty entering other treatment services until such time as their current high level of drunkenness was reduced. Generally speaking, the client group represented the most serious alcohol dependent individuals presenting for treatment.

### Treatment procedure

Each daily session was held from 3pm to 3.30pm, Monday to Friday over two successive weeks. On the Friday prior to starting, all clients were given simple advice on cutting down, such as replacing some stronger drinks with weaker ones, delaying the first drink by 30–60 minutes each day, drinking only enough to stop withdrawal symptoms and not drinking to get drunk. They were also given a 24 hour drink diary, which was to be completed from 4pm on Sunday. Clients were advised to drink as they normally would up to Monday when the group began and reduction in alcohol consumption could begin under closer supervision. The drink diary ran from 4pm to 3pm the next day to cover the period between the groups. Each group comprised a maximum of four clients.

On the first day, clients were expected to produce the drink diary cataloguing all alcohol-containing drinks in the previous 24 hours. Each client was breathalysed and this reading was shared with the other clients in the group. The alcohol consumed was converted into UK units for standardisation (Note that 1 UK unit of alcohol  =  10 millilitres or 8 grams of alcohol). Breath alcohol readings were taken with a Lion 400 Alcometer, which was calibrated monthly in accordance with the manufacturer's recommendations.

Discussion during group sessions focused on reduced day-by-day drinking quantities and on strategies to achieve a lower BrAC reading at the same time on the next day. Specifically, clients reported their subjective state of withdrawal or drunkenness and this report had a bearing on the mutually agreed unit consumption over the next 24 hours. Clients who had reduced too rapidly and showed marked signs of withdrawal discomfort were advised to drink only enough to quell the symptoms shortly after leaving the group session. Such clients were advised to take alcohol in doses of around 2 units, to wait half an hour and to reassess how they felt. If they continued to experience withdrawal symptoms beyond what they felt to be manageable, then they were advised to consume another 2 units of alcohol, and so on. Clients were advised to stop taking alcohol once the withdrawal discomfort became bearable. Conversely, if clients appeared to be, or reported feeling “drunk”, the same strategy was advised, that is, to drink only so much as to titrate between withdrawal discomfort and drunkenness until the next session.

At the end of each session, each client was given another 24-hour drink diary, and the previous one was returned to the client so that the previous drink diaries could act as a benchmark on which to base drinking over the next 24 hours. This was repeated over a period of ten working days. Clients who attained a zero BrAC reading on any session were expected to continue their attendance until the end of the week and then join the wider therapeutic day-hospital programme on the following Monday.

## Results

### Clients

Of the sample, 17.2% reported alcohol withdrawal related seizures on prior occasions, either during self-elected withdrawal, or during another treatment episode. By contrast, during the current self-paced alcohol withdrawal program, only 0.5% of clients reported a seizure, suggesting the program largely avoided this harmful consequence of withdrawal. The severity of alcohol dependence score [6] of clients, measured at initial clinical assessment, was 39.5 (standard deviation std = 9.8) indicating a classification of severe alcohol dependence in the sample as a whole. The sample had a mean age of 41.7 (std = 10.0), comprised 77.5% males, 28.0% were married, 17.5% were employed, 11.8% had diagnosed liver disease including cirrhosis and pancreatitis, 5.2% reported concurrent substance misuse, and 2.7% reported dual diagnosed of mental illness.

### Attrition

**Figure 1:**
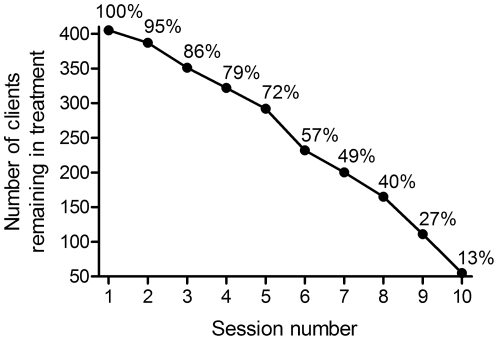
Number of clients attending 1–10 possible sessions of treatment, with floating numbers showing the percentage of clients out of the total sample of 405.

Each client's breath alcohol and diary reported units were ordered sequentially across the sessions they attended, into first, second, third, etc., through to tenth. Figure 1 shows the number clients, out of a total of 405 that attended 1–10 sessions. It is clear that there was substantial attrition of clients across the 10 possible sessions, from 100% to 13% by session 10. The number of sessions attended was not associated with either alcohol units per week reported at clinical screening, severity of alcohol dependence score [6], breath alcohol or diary reported units obtained at the first measurement, *r*s < .04, *p*s > .05.

### Mean breath alcohol

**Figure 2:**
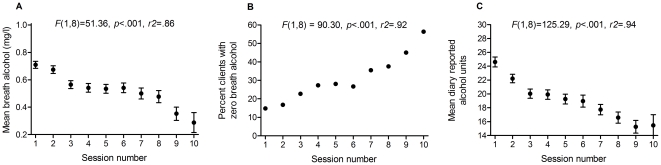
Mean breath alcohol scores (A), percent clients returning a zero breath alcohol score (B), and mean diary reported UK alcohol units (C) for clients who attended each of the 10 sessions. Note that 1 UK unit of alcohol  =  10 millilitres or 8 grams of alcohol.

To examine the overall decline in BrAC readings, averages for each session were obtained across all clients who attended that session, and plotted in Figure 2A. There was a significant linear fit to these averages, *F*(1,8) = 51.36, *p*<.001, *r^2^* = .86. Paired contrasts indicated that BrAC readings were significantly reduced relative to session 1, at session 3 onwards *F*s > 21.52, *p*s < .001, but not at session 2, *F*(1,386) = 1.75, *p* = .19.

### Percent clients achieving zero breath alcohol

One objective of treatment was to enable clients to achieve a zero BrAC reading to qualify for further treatment. In accordance with this objective, Figure 2B shows that the percentage of clients providing a zero BrAC score increased linearly across sessions, *F*(1,8) = 90.30, *p*<.001, *r^2^* = .92.

### Diary reported alcohol units

Finally, the average diary units obtained at each session, collapsed across the entire sample of clients who contributed data, showed a significant linear decline, *F*(1,8) = 125.29, *p*<.001, *r^2^* = .94, as shown in Figure 2C. Paired contrasts indicated that reported diary units were significantly reduced relative to session 1, from session 2 onwards *F*s > 11.56, *p*s ≤ .001.

### Relationship between BrAC and diary reported consumption

**Figure 3:**
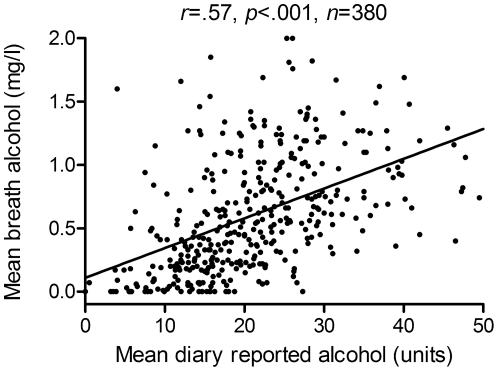
Scatterplot showing the relationship between breath alcohol and diary reported alcohol units. Note that 1 UK unit of alcohol  =  10 millilitres or 8 grams of alcohol.

There is a question concerning the accuracy of the self-reported drink diaries. BrAC and diary reported units showed comparable linear declines across sessions, suggesting a broad correspondence between these measures. To evaluation the relationship between these measures, BrAC scores and diary reported units were averaged across all sessions, and these means were subjected to Spearman's correlations. Figure 3 shows the scatterplot for this association and indicates that BrAC and diary reported units were correlated (note that the number of participants included in this correlation was smaller than the total number of clients because 25 clients did not supply drink diaries).

### Correlation or causation

The foregoing analyses indicated that increasing attendance was associated with a linear reduction in alcohol consumption. However, because this association is correlational, that is, there was no ‘placebo’ control group, it is impossible to say whether the treatment protocol caused the reduction in alcohol consumption, or whether a third variable, that was confounded with greater attendance, mediated the reduction in alcohol consumption. The most trivial of these ‘non-causal’ accounts is the possibility that across sessions, clients with the greatest baseline alcohol consumption showed higher rates of attrition, such that as sessions proceeded, there was an increasing concentration of clients with lower alcohol consumption. However, as noted earlier, there were no significant correlations between baseline alcohol consumption and attendance, suggesting that selective attrition of higher drinkers was not responsible for the relationship between attendance and reductions in alcohol consumption.

The second non-causal account of the relationship between attendance and reduction in alcohol consumption was that the simple passage of time, rather than the group intervention, was responsible for the reduction in alcohol consumption. Although this trivial account is always a logical possibility in explaining treatment effects that lack a control group, the force of this argument is weakened by the protracted nature of clients' alcohol dependence. If time alone led to abstinence, clients should have quit using alcohol long before they presented for treatment. Accordingly, we would argue that the intervention was the causal factor mediating the reduction in alcohol consumption.

## Discussion

The principle finding of the study was that attendance at treatment sessions was associated with decline in alcohol consumption reflected in mean breath alcohol recordings, the percentage of clients achieving a zero breath alcohol reading, and diary reported alcohol units. Although the absence of a control group exposes this association to ‘non-causal’ explanations, such as selective attrition of higher drinkers or the simple passage of time, the absence of direct evidence supporting these accounts speaks in favour of the treatment protocol playing a causal role in reducing alcohol consumption.

In absolute terms, the treatment protocol was associated with an increase in the percentage clients returning a zero breath alcohol score from 15% at baseline, to 35% at the final session, across all clients. Moreover, this percentage varied as a function of how many sessions clients attended, increasing up to 56% in clients who attended all 10 sessions (see Figure 2B). Despite this reduction in drinking, only 0.5% of clients reported a withdrawal related seizure, yet 17.2% of clients had reported seizures in other circumstances prior to treatment. Our conclusion, therefore, is that the un-medicated, self-paced alcohol withdrawal procedure described here is an effective strategy for reducing alcohol consumption, that avoids some of the adverse consequences of acute alcohol detoxification [1].

If attendance at treatment was causal in mediating the reduction in alcohol consumption, then strategies designed to enhance attendance should increase the effectiveness of the treatment. Consistent with this claim, contingency management has been shown to significantly increase both compliance and the effectiveness of treatment protocols for substance misuse [7], and is being offered by an increasing number of substance abuse treatment service providers [8]. The current finding of an association between attendance and alcohol reduction, therefore, strongly justifies the incorporation of contingency management into the gradual alcohol reduction protocol.

It is possible that un-medicated, out-patient, gradual alcohol reduction interventions teach clients to regulate their drinking behaviour in their home environment, and enhance self-efficacy by allowing attribution of success to self-control rather than to a pharmacological intervention or constraints at being in an in-patient environment [9], [10]. Indeed, in contrast to the one-drink-one-drunk dogma, Heather and Robertson [11] have argued that “drinking behaviour itself should be the main focus of concern in treatment and must not only be allowed to occur but positively encouraged so that associated emotional responses and motivations for drinking can be understood and a new pattern of behaviour developed.” (see also; [12]). Whatever precise psychological mechanisms are engaged by the current treatment, the study suggests that at least short term control over drinking can be achieved with a therapeutic intervention that allows drinking to take place in the home environment.
